# Supplementary material for: Characterisation of the ARF Gene Family in Salicaceae and Functional Analysis of PeARF18 in Heteromorphic Leaf Development of Populus euphratica
Source: Int J Mol Sci. 2025 Dec 28;27(1):335. doi: 10.3390/ijms27010335 (PMC12785610; doi:10.3390/ijms27010335)
Supplement: Supplementary file 1 [file ijms-27-00335-s001.zip › Supplementary Materials.pdf]

Supplemental Materials：

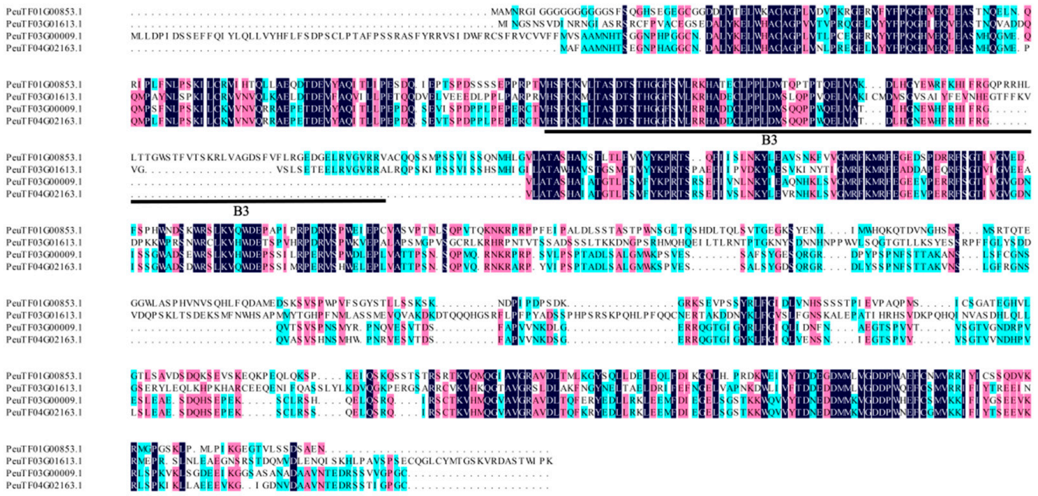

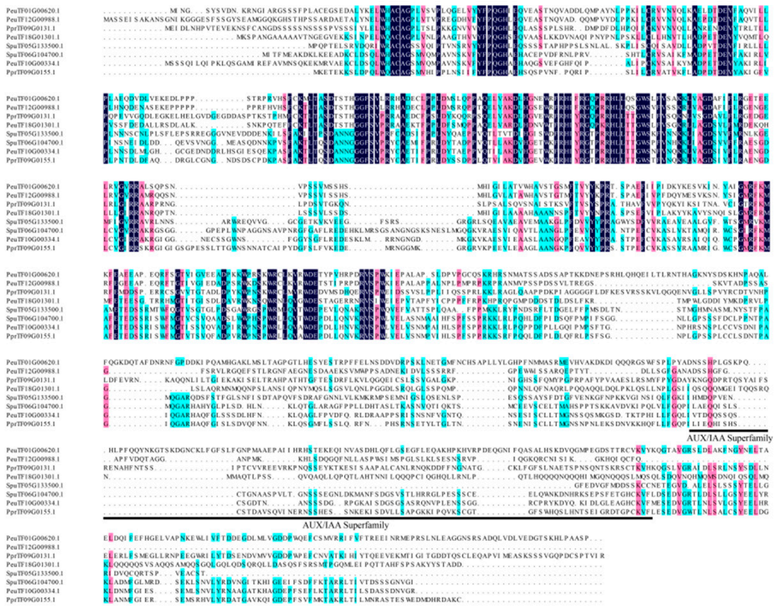

Figure S1: Multiple sequence alignment of conserved domains within ARF proteins from three Salicaceae species.

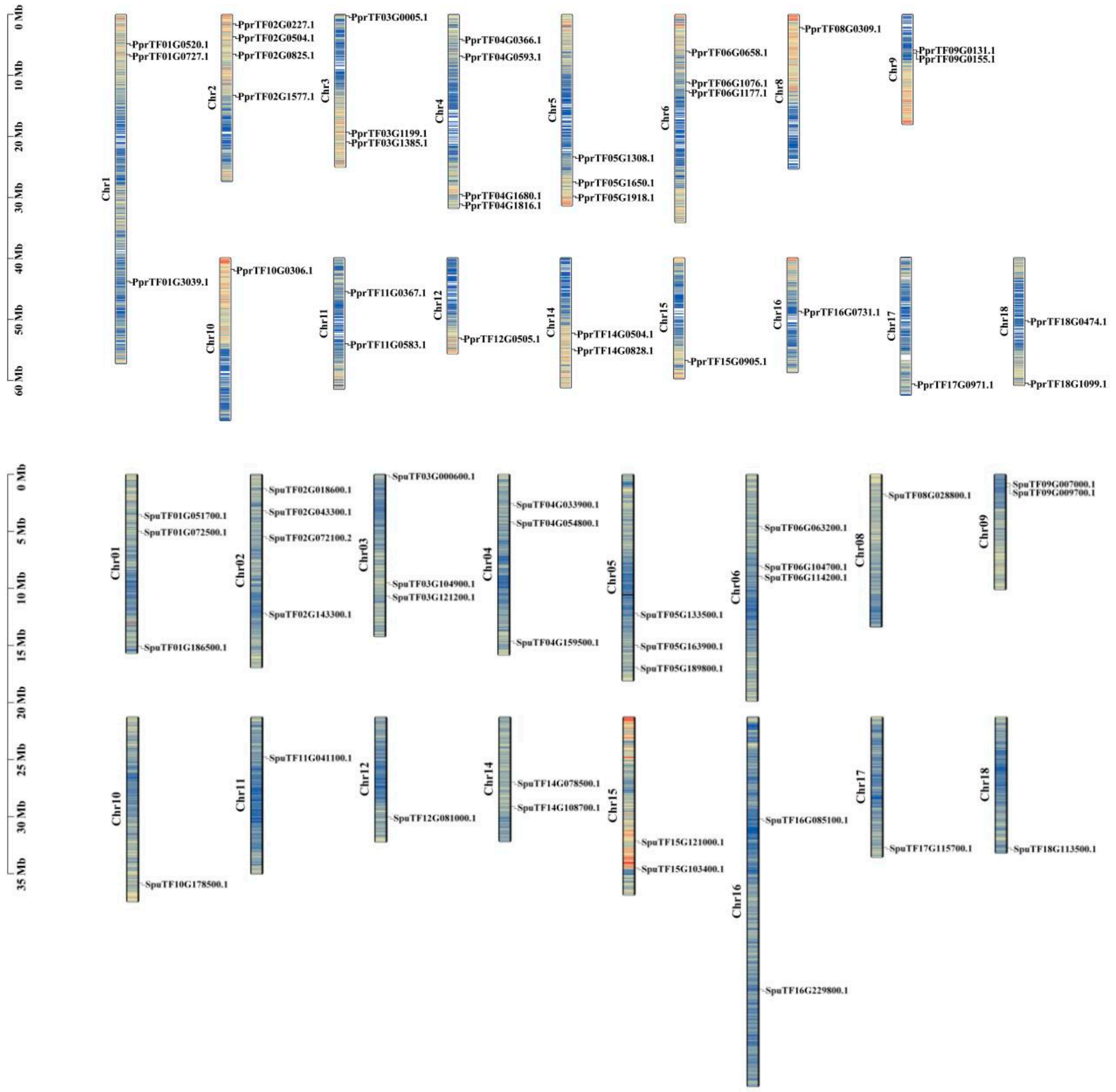

Figure S2: Chromosomal localization of *ARF* gene family members in *Populus pruinosa* and *Salix purpurea*

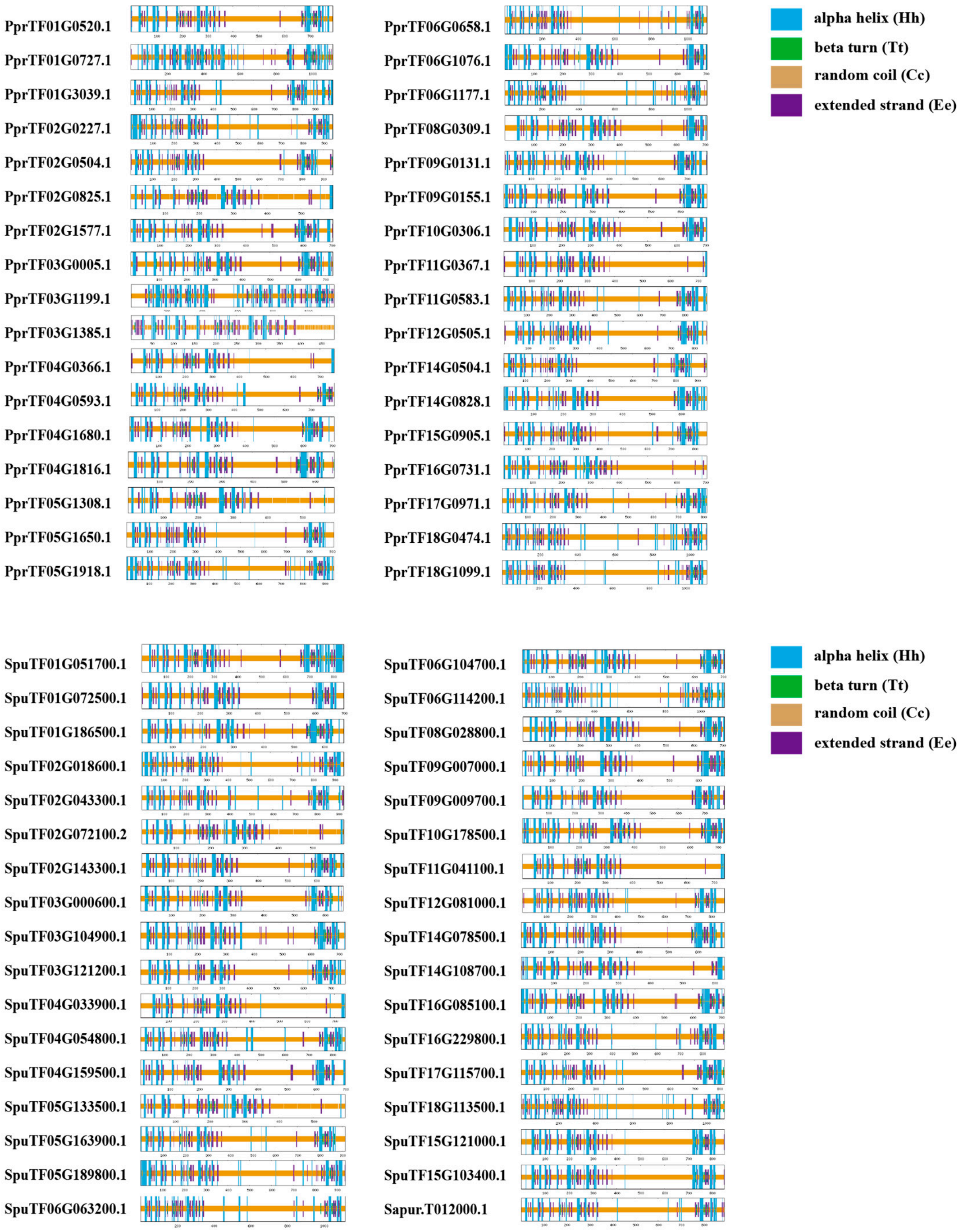

Figure S3: The secondary structure of proteins of *ARF* gene family members in *Populus pruinosa* and *Salix sinopurpurea.*
